# Clinical and ultrasonography evaluation of thyroid tumor screening in symptomatic patient of Bajulmati primary care center, Banyuwangi, East Java, Indonesia

**DOI:** 10.1097/MD.0000000000032546

**Published:** 2022-12-30

**Authors:** Rosy Setiawati, Tri Wulanhandarini, Fierly Hayati, Dyah Erawati, Merlin Guntur Jaya, Andi Ahmad Thoriq, Triana Mediyawati Wijaya, Galih Nur Ismiyati, Dyan Wahyu Kusumaningrum, Belinda Koesmarsono, Agnes Triana Basja, M. Ikhsan Nugroho, Silvi Yuliana, Syadza Zahrah Shedyta, Hendra Boy Situmorang

**Affiliations:** a Radiology Department, Universitas Airlangga, Faculty of Medicine, Dr. Soetomo Academic General Hospital, Surabaya, Indonesia.

**Keywords:** primary health care, screening, thyroid cancer, ultrasonography

## Abstract

This study aims to assess the prevalence, clinical, and ultrasonography (US) in thyroid screening in healthy subjects with general symptoms of thyroid abnormality in low iodine intake in Bajulmati primary care center, East Java Indonesia. We retrospectively reviewed US thyroid examination of 74 subjects with symptoms of mass in the neck, shaky, sleep difficulties, over sweating, and chronic fatigue on September 15^th^, 2021. Following the WHO guidelines, subjects also underwent physical examination in which the result were classified into 3 categories, that is, no palpable nor visible goiter, palpable but no visible goiter, as well as palpable and visible goiter. We evaluate US thyroid characteristics following Korean Society of Thyroid Radiology guidelines. Image analysis was reviewed by 4 general radiologists with 2 to 13 years’ experience. Categorical variables were compared using chi-squared or Fisher exact tests. Correlation between variables was measured with gamma statistics. Statistical analyses were conducted using IBM SPSS Statistics 23.0. A *P*-value < .05 was considered to indicate statistical significance. Of the 74 subjects, 32 (43.2%) show abnormalities. Statistical analysis showed no significant differences in the result of thyroid US in subjects with complaint fatigue (*P* = .464), insomnia (*P* = .777), over sweating (*P* = .158), and tremor (*P* = .778), but there were significant differences with the complaint of mass in the neck (*P* = .008). Furthermore, there was also a strong correlation between goiter palpation and US thyroid result (*R* = 0.773, *P* = .00). We conclude there were significant differences in US result of patients with and without complaint of mass in the neck. We also found a strong correlation between goiter palpation and US examination. Clinical findings, laboratory examination, cytology and molecular markers, patients’ age, nodules size, and ultrasound features should be considered for the treatment planning.

## 1. Introduction

Thyroid cancer in 2020 had an incidence rates of 10.1 per 100.000 women and 3.1 per 100,000 men with age-standardized mortality rates 0.5 per 100.000 women and 0.3 per 100.000 men globally.^[1]^ There is increase of incidence reported with findings: nearly all registered increase is limited to papillary histotype which is the least aggressive variant of thyroid cancer, and it is more marked in population groups with higher socioeconomic standard and better healthcare access.^[2]^ The true thyroid cancer increase might be due to environmental factors, female genetic and hormonal background of people who are more prone to thyroid diseases.^[3]^

The early finding of the malignant thyroid cancer can help decreasing mortality, reduce morbidity and to avoid unnecessary tests and surgery in benign nodule, hence the nodule detection is crucial.^[4]^ Routine examination of thyroid include physical examination inspection and palpation of the neck. A more detailed, noninvasive, inexpensive could be achieved with ultrasonography.^[5]^ Ultrasonography (US) becomes a choice for the screening tools in thyroid abnormality for its non-invasiveness and its availability.^[6]^ The advances of the US had resulted in the identification of the thyroid nodules whether in the asymptomatic or symptomatic patient.^[7]^ Thyroid nodules detected by the US were 65%, while in the palpations were 4%.^[8]^

Thyroid nodules may depend on several factors, including low iodine intake in the area with iodine insufficiency.^[9]^ Iodine intake is a crucial determinant of the prevalence of thyroid abnormality in a population where thyroidal hyperplasia, hypertrophy, and nodules formation may happen within months of the iodine deficiency from the free radical oxygen mutagenic effects.^[10]^ Low iodine intake may increase the thyroid-stimulating hormone (TSH) stimulation and causing increased thyroid cell responsiveness to TSH, thus resulting in thyroid cell epidermal growth factor beta 1 production and increased angiogenesis related to promotion of tumor growth.^[11]^ East Java is one of Indonesia’s regions with low median urinary iodine concentration (<150 *µ*f/L), showing deficiency of iodine in the East Javanese population.^[12]^

Currently, no studies had evaluated the use of ultrasonography screening to provide clear guidance and to improve clinicians’ judgment, especially in Indonesia with high rates of low iodine intake.^[13]^ Therefore, early detection of thyroid abnormality could benefit the treatment in the future. This study aims to assess the prevalence, clinical, and US in thyroid screening in healthy subjects with general symptoms of thyroid abnormality in Bajulmati primary care center, East Java Indonesia.

## 2. Materials and Methods

### 2.1. Patients

This retrospective study was approved by the ethical board of our institution, and the necessity for informed consent for research was waived. A total of 76 consecutive symptomatic subjects underwent screening thyroid US at the primary care center Bajulmati with community services on September 15^th^, 2021. We include subjects with symptoms of mass in the neck, shaky, sleep difficulties, over sweating, and chronic fatigue. There were 2 subjects with a history of thyroid surgery excluded from this study. The subjects were asked about their symptoms then underwent neck palpation examination. Following the WHO guidelines, subjects were then separated into 3 classifications: subjects with no palpable nor visible goiter, palpable but no visible goiter, as well as palpable and visible goiter.^[14]^

### 2.2. US imaging

US thyroid examination was performed at the primary care center in Bajulmati Banyuwangi Indonesia using portable US equipment Samsung HM70A and Midray D10 with linear transducer by 4 board-certified radiologists. Based on the recommendation by the Korean society of thyroid radiology, thyroid nodules were described by the features of the nodule. The features mentioned in our study include composition, echogenicity, margins, axis, calcification, size. The composition includes cystic (>90% of the cystic portion) or predominantly cystic (>50% of the cystic portion and < 90% of the cystic portion) nodules with reverberating artifacts and spongiform nodules. The echogenicity includes hypoechoic, isoechoic, and hyperechoic. The size of the nodule can be > 1 cm, > 2 cm. The margin mentioned could be smooth, spiculated, and ill-defined. While shapes mentioned were ovoid to round, taller than wider, and irregular.

### 2.3. Imaging analysis, reference standards, and statistical analysis

Image analysis was reviewed by 4 general radiologists with 2 to 13 years’ experience. In multiple nodules, the most suspicious nodules were the 1 to be described. Discrepancies in US results will be resolved by the opinion of the most experienced radiologist. Categorical variables were compared using chi-squared or Fisher exact tests, while the correlation between variables was measured with gamma statistics. Statistical analyses were conducted using IBM SPSS Statistics 23.0. A *P*-value < 0.05 was considered to indicate statistical significance.

## 3. Results

A total of 74 subjects included in this study were 68 women and 6 men with a mean age of 42.71 years (range from 1 year to 87 years). Among them, 15 subjects (20.3%) with complaint of tremor, 15 subjects (20.3%) with a complaint of over sweating, 16 subjects (21.6%) with a complaint of insomnia, 26 subjects (35.1%) with a complaint of chronic fatigue, and 20 subjects (17%) with a complaint of a neck mass. (Table 1)

**Table 1:** Patients’ demography.

Patient demography	
Age (range) (yr)	42.71 (0.88–87)
Gender	
Female	68 (91.9%)
Male	6 (8.1%)
Patients’ complaint	
Palpitation	22 (29.7%)
Shaky	15 (20.3%)
Over sweating	15 (20.3%)
Insomnia	16 (21.6%)
Chronic fatigue	26 (35.1%)
Neck mass	20 (17.0%)
Neck physical examination	
Not palpable nor seen	52 (70.3%)
Palpable but not seen	5 (6.8%)
Seen enlarged	17 (23%)

Among the included subjects, 32 subjects (43.2%) have nodule abnormality. The nodule involvement were 17 subjects (23%) unilateral and 15 patients (20.3%) bilateral. Subjects with single nodule were 17 subjects (23%) while with multiple nodules were 15 subjects (20.3%). The shape of the nodules for 30 subjects (40.5%) was ovoid to round or wider than taller while 2 subjects (2.7%) were taller than wider. The contents of the right thyroid nodules were solid in 6 subjects (8.1%), predominant solid in 6 subjects (8.1%), predominant cystic in 7 subjects (9.5%), cystic in 5 subjects (6.8%), and spongiform in 3 subjects (4.1%). While the contents of the left thyroid nodules were solid in 1 subject (1.4%), predominant solid in 8 subjects (10.8%), predominant cystic in 7 subjects (9.5%), cystic in 3 subjects (4.1%), and spongiform in 2 subjects (2.7%). The echogenicity of the nodules in the right thyroid was hypoechoic in 13 subjects (17.6%), isoechoic in 2 subjects (2.7 %), hyperechoic in 5 subjects (6.8%), and mixed echo in 2 subjects (2.7%). While in the left side, the nodules were hypoechoic in 17 subjects (23%), isoechoic in 3 subjects (4.1%), hyperechoic in 5 subjects (6.8%), and mixed echo in 1 subject (1.4%). The margin of the nodules in the right side of the thyroid were smooth in 24 subjects (32.4%), ill-defined in 3 patients (4.1%) while in the left side of the thyroid 19 subjects (25.7%) were smooth and 2 subjects (2.7%) were ill-defined. Subjects with nodules calcification shaped stippled, fine coarse and nonlinear were 2 subjects (2.7%), and 2 other subjects (2.7%) were shaped curvilinear, smooth margin (Table 2).

**Table 2:** US Features of screening-detected thyroid cancer.

US result	
Normal	42 (56.8%)
With nodul	32 (43.2%)
Involvement	
Unilateral	17 (23%)
Bilateral	15 (20.3%)
Amount of nodul	
Single	17 (23%)
Multiple	15 (20.3%)
Shape of the nodul	
Ovoid to round	30 (40.5%)
Taller than wider	2 (2.7%)
Content of the right thyroid nodul	
Normal	47 (63.5%)
Solid	6 (8.1%)
Predominant solid	6 (8.1%)
Predominant cystic	7 (9.5%)
Cystic	5 (6.8%)
Spongioform	3 (4.1%)
Content of the left thyroid nodul	
Normal	53 (71.6%)
Solid	1 (1.4%)
Predominant solid	8 (10.8%)
Predominant cystic	7 (9.5%)
Cystic	3 (4.1%)
Spongioform	2 (2.7%)
Echogenicity of right thyroid nodul	
Normal	53 (71.6%)
Hypoechoic	13 (17.6%)
Isoechoic	2 (2.7%)
Hyperechoic	5 (6.8%)
Mixed	2 (2.7%)
Echogenicity of left thyroid nodul	
Normal	47 (63.5%)
Hypoechoic	17 (23.0%)
Isoechoic	3 (4.1%)
Hyperechoic	5 (6.8%)
Mixed	1 (1.4%)
Margin of the right mass	
Normal	47 (63.5%)
Smooth	24 (32.4%)
Ill defined	3 (4.1%)
Margin of the left mass	
Normal	53 (71.6%)
Smooth	19 (25.7%)
Ill defined	2 (2.7%)
Mass Calcification	
No calcification	70 (94.6)
Stipplen, fine coarse, non linear	2 (2.7%)
Curvelinear, smooth margin	2 (2.7%)

US = ultrasonography.

Statistical analysis showed there were significant differences in US result of patients with and without complaint of mass in the neck (*P* = .008). (Table 3) There was also a significant strong correlation between goiter palpation and US thyroid result (*R* = 0.773, *P* = .00). (Table 4)

**Table 3:** Comparison of patient complaint in normal patient and in patient with nodul.

	Normal (n = 42)	Nodul (n = 32)	*p*-value
Patient complaints			
Mass in the neck	6	14	< .01
Fatigue	13	13	> .05
Insomnia	10	6	> .05
Over sweating	6	9	> .05
Shaky	8	7	> .05

**Table 4:** Correlation of neck physical examination to US result.

Goitre palpation	Normal (n = 42)	Nodule (n = 32)	Coef. Correlation	*P* value
No palpable/visible mass	37	15	0.773	< .01
Palpable but not visible mass	2	3		< .01
Palpable and visible mass	3	14		< .01

US = ultrasonography.

## 4. Discussion

Thyroid incidentalomas can be found in the imaging study for non-thyroid neck disease, on the US in the screening procedure, or in the histopathologic examination in the surgical specimens for non-nodular disease. The prevalence data from ultrasonographic studies were revealed ranging from 19% to 46% in the general population.^[15]^ The result was similar to this study of 43.2% (32 subjects) nodule detection among subjects with symptoms. Similar results of incidentalomas findings showed in the Choi study that was 37%.^[16]^ That being said, study of 2079 patients in Korea showed high sensitivity and specificity 96.2% and 51.7%, respectively.^[17]^ In the 253 randomly selected adults, 5.1% had abnormality of the neck with sensitivity and specificity to detect thyroid nodules were 11.6% and 97.3%, respectively.^[18]^ Of 1845 subjects, one of the study show cancer rate detection of 1.6% with sensitivity and specificity 100% and 98.7%, respectively, showing good diagnostic performance of thyroid screening US.^[19]^

The value of thyroid US study is still a debate due to several reasons including overdiagnosis which potentially cause overtreatment.^[20]^ This may lead to unnecessary procedures and expose the patient to the treatment side effect.^[21]^ The increased incidence and low mortality interpreted as a result of overdiagnosis.^[22]^ Moreover, study from Jun et al shows no difference in the mortality between the patient who went through screening and those who don’t.^[23]^ The risk of overdiagnosis could be diminished by taking account into other factor such as size of thyroid nodules, presence of lymphadenopathy, cytology and molecular markers results, TSH measurement, US characteristics, patients’ age, family history of thyroid cancer, and history of head and neck irradiation.^[6]^

Routine screening population of low risk for thyroid cancer individual were unlikely to be cost effective and more useful in higher-risk individuals such as patient with radiation exposure, positive family history, lesser than 14 years or greater than 70 years of age with palpable thyroid nodule, and with suspicious clinical signs.^[24]^ With the advancement of the artificial intelligence, some models can be used to minimize false positives although with the cost of increasing false negative.^[25]^ Long-term controlled trials are a necessity to be performed to study the efficacy of US thyroid screening.

Chen et al stated the proper use of ultrasonography is when there are clinically supported features such as examination finding of a palpable thyroid nodule, large thyroid or a goiter, thyroid nodule findings from another imaging test, and new-onset hoarseness or compressive symptoms^.[26]^ Even so, the definition of inappropriate use of thyroid ultrasound still varies. It could be due to deemed unnecessary by reviewing endocrinologists, functional disease or nonspecific symptoms without specifying the presence of a palpable mass/ nodule, hypothyroidism, no history of thyroid nodule or neck irradiation.^[27]^ In this study, there was a significant difference in the symptoms of mass in the neck of the subjects (*P* = .008). Moreover, there was a strong correlation between goiter palpation dan US thyroid result (*R* = 0.773, *P* = .00). This result is in line with a study by Germano et al in which the incidentalomas were found almost as frequently as the palpable nodules demonstrating the important role of imaging.^[28]^

Arpana et al showed USG study results of texture, size, margin, echogenicity, and vascularity are important factors for predicting thyroid malignancy but are not to be used independently as a screening tool^.[29]^ The combination of suspicious findings can help differentiate nodules that may require fine needle aspiration (FNA).^[30,31]^

Iodine deficiency was one of the risk factors that must be taken into account in associating with increased rates of thyroid diseases.^[6]^ Indonesia was included in the category of adequate iodine nutrition (UIC 100–299 *µ*g/L) based on national iodine status in 2014 through median urinary iodine concentration.^[32]^ The distribution of iodine status in Indonesian province, however, was not equally adequate. The risk of nodular goiter is only increased at the low intake due to the promotion of cell growth and DNA mutagenesis causing clusters of autonomous thyrocytes.^[10,32,33]^ However, iodine enrichment has been associated with increased thyroid cancer and shift from the follicular to papillary histotype.^[34]^

There are several limitations in our study. Our study includes a small number of samples due to the short duration of the community services and the retrospective nature. Secondly, we did not perform further examination such as FNA or treatment of the subjects. There was no blinding in the subjects, and the distribution of the patients is mostly female due to the timing of the examination. The study was also being conducted in the rural area at a primary care facility, thus there was no laboratory facility to support this study. One of the goals of this study was to help screening of thyroid abnormality, especially at rural area without laboratory facility to predict thyroid function without the need of thyroid tests such as biochemical marker. This is also showed the importance of this study, since the result showed that there were significant differences in US result of patients with and without complaint of mass in the neck. We also found a strong correlation between goiter palpation and US examination. Therefore, it is important to do US examination for patient with complaint of mass in the neck and with goiter. Ultrasound can also be an alternative to laboratory limitation in the rural area because the modality of ultrasound is mobile, cheap, and noninvasive. Our suggestion for future study is there should be a screening thyroid study on the nation scale with a larger sample and correlating with the FNA or biopsy results and biochemical marker of thyroid function. The mortality rate of the thyroid cancer should be take into consideration to avoid overdiagnosis.

We conclude there were significant differences in US result of patients with and without complaint of mass in the neck. We also found a strong correlation between goiter palpation and US examination. Although US examination proves to be beneficial in nodule thyroid detection, other factors namely clinical, laboratory examination, cytology and molecular markers, patients’ age, nodules size, and ultrasound features should be held into account before taking the further step of treatment.

## Acknowledgments

The authors would like to thank all who have contributed to the process and completion of this report, including the teaching staffs and fellow residents of Department of Radiology of Faculty of Medicine, Universitas Airlangga, Dr Soetomo Academic General Hospital, Surabaya, Indonesia. We also want to thank Bajulmati primary care center, Banyuwangi, East Java, Indonesia.

## Author contributions

**Conceptualization:** Rosy Setiawati, Tri Wulanhandarini, Fierly Hayati, Dyah Erawati, Merlin Guntur Jaya, Agnes Triana Basja.

**Data curation:** Rosy Setiawati, Fierly Hayati, Dyah Erawati, Merlin Guntur Jaya, Andi Ahmad Thoriq, Triana Mediyawati Wijaya, Galih Nur Ismiyati, Dyan Wahyu Kusumaningrum, Belinda Koesmarsono, M. Ikhsan Nugroho, Silvi Yuliana, Syadza Zahrah Shedyta, Hendra Boy Situmorang.

**Investigation:** Rosy Setiawati, Fierly Hayati, Ahmad Thoriq, Triana Mediyawati Wijaya, Galih Nur Ismiyati, Dyan Wahyu Kusumaningrum, Belinda Koesmarsono, Agnes Triana Basja, M. Ikhsan Nugroho, Silvi Yuliana, Syadza Zahrah Shedyta, Hendra Boy Situmorang.

**Methodology:** Rosy Setiawati, Merlin Guntur Jaya.

**Supervision:** Dyah Erawati.

**Validation:** Rosy Setiawati.

**Writing – original draft:** Rosy Setiawati, Merlin Guntur Jaya.

**Writing – review & editing:** Rosy Setiawati, Tri Wulanhandarini, Merlin Guntur Jaya.
